# The Safety and Efficacy of Lansoprazole plus Metoclopramide among Neonates with Gastroesophageal Reflux Disease Resistant to Conservative Therapy and Monotherapy: A Clinical Trial

**DOI:** 10.1155/2021/3208495

**Published:** 2021-01-15

**Authors:** Peymaneh Alizadeh Taheri, Elahe Validad, Kambiz Eftekhari

**Affiliations:** ^1^Department of Neonatology, Tehran University of Medical Sciences, Bahrami Children Hospital, Tehran, Iran; ^2^Department of Pediatrics, Tehran University of Medical Sciences, Bahrami Children Hospital, Tehran, Iran; ^3^Department of Gastroenterology, University of Medical Sciences, Bahrami Children Hospital, Tehran, Iran

## Abstract

**Background:**

Gastroesophageal reflux disease (GERD) is one of the most common problems in neonates. The main clinical manifestations of neonatal GERD are frequent regurgitation or vomiting associated with irritability, crying, anorexia or feeding refusal, failure to thrive, arching of the back, and sleep disturbance.

**Aims:**

The efficacy and safety of ranitidine plus metoclopramide and lansoprazole plus metoclopramide in reducing clinical GERD symptoms based on I-GERQ-R scores in neonatal GERD resistant to conservative and monotherapy. *Study Design*. This study was a randomized clinical trial of term neonates with GERD diagnosis (according to the final version of the I-GERQ-R), resistant to conservative and monotherapy admitted to Bahrami Children Hospital during 2017-2019. Totally, 120 term neonates (mean age 10.91 ± 7.17 days; girls 54.63%) were randomly assigned to a double-blind trial with either oral ranitidine plus metoclopramide (group A) or oral lansoprazole plus metoclopramide (group B). The changes of the symptoms and signs were recorded after one week and one month. At the end, fifty-four neonates in each group completed the study and their data were analyzed.

**Results:**

There was no significant difference in demographic and baseline characteristics between the two groups. The response rate of “lansoprazole plus metoclopramide” was significantly higher than “ranitidine plus metoclopramide” (7.44 ± 3.86 score vs. 9.3 ± 4.57 score, *p* = 0.018) after one week and (2.41 ± 3.06 score vs. 4.5 ± 4.12 score, *p* = 0.003) after one month (primary outcome). There were no drug adverse effects in either group during intervention (secondary outcome).

**Conclusions:**

The response rate was significant in each group after one week and one month of treatment, but it was significantly higher in the “lansoprazole plus metoclopramide” group compared with the “ranitidine plus metoclopramide” group. The combination of each acid suppressant with metoclopramide led to a higher response rate in comparison with monotherapy used before intervention. This study has been registered at the Iranian Registry of Clinical Trails (RCT20160827029535N3).

## 1. Introduction

Gastroesophageal reflux (GER) is a physiologic reverse passage of gastric contents into the esophagus with or without regurgitation and/or vomiting [1, 2]. It is commonly observed during the first year of life and almost happens in 65% of infants at the age of 3–6 months [2]. Gastroesophageal reflux disease (GERD) occurs when troublesome symptoms or conditions complicate the physiologic GER [1–4]. The main clinical manifestations of neonatal GERD include frequent regurgitation or vomiting associated with irritability, excessive crying, anorexia or feeding refusal, hematemesis, failure to thrives, Sandifer syndrome, anemia, sleep disturbance, coughing, choking, wheezing, stridor, apnea spells, recurrent pneumonia aspiration, recurrent otitis media, or upper respiratory tract symptoms [1, 5, 6].

Malformations of the central nervous system (CNS) and gastrointestinal tract (e.g., esophageal atresia and congenital diaphragmatic hernia), a positive family history of GERD, cystic fibrosis, hiatal hernia, family history of severe GERD, neurologic impairment, drugs (e.g., sedatives and muscle relaxants), and prematurity are factors that increase the risk of GERD in infants [3, 4].

Acid suppressants, including histamine-2 receptor antagonists (H2RAs) and proton pump inhibitors (PPIs), have been used as a pharmacologic treatment of pediatric GERD to induce symptomatic relief and normal growth and to prevent its complications and recurrence [7]. According to the recent guidelines, a 2-4-week trial of a PPI or H2RA is recommended for infants with significant regurgitation accompanied with symptoms such as unexplained feeding problems, troubled behavior, and poor weight gain [4]. PPIs (e.g., lansoprazole) facilitate gastric emptying and inactivate H+/K+-ATPase in the gastric parietal cells canaliculi, leading to inhibition of gastric acid production and secretion [8, 9]. PPIs induce a more vigorous inhibition of acid secretion, have a longer duration of action, and cause fewer complications and no tachyphylaxis compared to H2RAs [5, 10]. Prokinetics increase the LES tone and gastric emptying [11]. Among prokinetics, although metoclopramide may induce irritability, drowsiness, oculogyric crisis, dystonic reaction, apnea, and emesis in infants, these adverse reactions are only induced with prolonged or high-dose metoclopramide exposure [12]. On the other hand, two other prokinetics including domperidone and cisapride may induce cardiac arrhythmia and are prohibited to be used in the USA [13, 14]. Macrolides are known as prokinetics, but they may also induce cardiac arrhythmia in long-term exposure [15]. Totally, metoclopramide is a safe prokinetic if it is administered with short-duration and low-dose amount, so we preferred to use it in this study.

There are still controversies about the management of neonatal GERD. To the best of our knowledge, very few clinical trials have compared the effectiveness of PPIs and H2RAs in pediatric GERD, especially in neonates and infants [16, 17]. Since no study has compared the efficacy and safety of metoclopramide plus ranitidine with metoclopramide plus lansoprazole in the management of neonatal GERD resistant to conservative therapy and monotherapy, this study was carried out.

## 2. Patients and Methods

This double-blind randomized controlled trial was conducted to compare the effectiveness of metoclopramide plus lansoprazole and metoclopramide plus ranitidine for GERD in term neonates.

### 2.1. Subjects

One hundred and twenty term neonates (postnatal age < 28 days, gestational age: 38-40 weeks) that presented to Bahrami Children's Hospital during 2016-2019 with a clinical diagnosis of GERD were enrolled in this study. The participants in both groups were fed with breast milk. The frequency of feeding was every two hours.

All patients were resistant to conservative therapy plus ranitidine or conservative therapy plus lansoprazole as the first line of treatment before intervention. The clinical improvement was <50% as defined as <50% reduction rate in the I-GERQ-R score (Table 1) before intervention. The conservative therapy included postural change, reduction of the feeding volume, and increasing the frequency of feedings (Table 1). Metoclopramide was added to ranitidine in patients of group A who had received ranitidine before intervention. On the other hand, metoclopramide was added to lansoprazole in patients of group B who had received lansoprazole before intervention.

The neonates with any significant underlying conditions (e.g., major congenital abnormalities and gastrointestinal or neurological disorders) or diseases (e.g., sepsis and cow's protein milk allergy), those who required invasive or noninvasive ventilation, and patients who administered any muscle relaxant or sedative medication were excluded from the study.

The number of participants was determined by a prospective power analysis, assuming a power of at least 80%, a 2-sided alpha of 0.05, and treatment response based on the studies of Springer et al. [18] and Famouri et al. [19].

### 2.2. Diagnosis

In this study, a diagnosis of GERD was made according to the final version of the I-GERQ-R and validity clinical score consisting of 12 items including the frequency, amount, and discomfort attributable to spit up (3 items), refusal or stopping feeding (2 items), crying and fussing (3 items), hiccups (1 item), arching back (1 item), and stopping breathing or color change (2 items). The items in the I-GERQ-R are summed, yielding a total score ranging from 0 to 42 with a cut point > 15 scores (Table 1) [20].

A significant rate of response to combination therapy also confirmed the diagnosis of GERD in each patient. Other diagnoses were ruled out based on the clinical manifestations of the patients, for example, if there were vomiting, apnea, or failure to thrive; clinical examination, lab tests, brain sonography, etc. were used to rule out sepsis, intraventricular hemorrhage, and other causes. The duration of conservative treatment and monotherapy was about 3-7 days each according to a careful balance of risks and benefits between the severity of clinical problems and the response rate. The term “resistant to conservative therapy and monotherapy” was applied when the clinical improvement was <50% as defined as <50% reduction rate in the I-GERQ-R score (Table 1) before intervention.

### 2.3. Trial

The study protocol was approved by the Research Ethics Committee of Tehran University of Medical Sciences (IR.TUMS.MEDICINE.REC.1396.3714). It was also registered in the Iranian Registry of Clinical Trails (RCT20160827029535N3). Written informed consent was obtained from parents or guardians of all infants before enrollment. They were explained about the study and procedures.

Mothers were assured of the confidentiality of their information, and they were also made to understand that participation was voluntary and participants could opt out at any stage of the study.

One hundred and twenty term neonates who met the inclusion and exclusion criteria were randomly assigned (in blocks of two per site) to a double-blind clinical trial to receive lansoprazole plus metoclopramide or ranitidine plus metoclopramide for a 30-day period. The random allocation sequence was generated by an independent statistician. The medical clinician, the researcher who collected the data, and the statist who analyzed the data were blind to the study.

Sixty neonates in group A received oral ranitidine 2 mg/kg/dose three times daily plus metoclopramide 0.15 mg/kg/dose three times daily, and sixty neonates in group B received oral lansoprazole 0.5 mg/kg/dose twice daily plus metoclopramide 0.15 mg/kg/dose three times daily. Before intervention, a checklist including demographic data (age, gender, birth weight, and weight at presentation) and GERD symptoms and signs according to I-GERQ-R scores was filled by a neonatologist (clinical researcher). The same neonatologist evaluated the clinical manifestations of patients according to I-GERQ-R scores after one week and one month to define whether improvement remains stable or continues to improve significantly.

**Figure 1:**
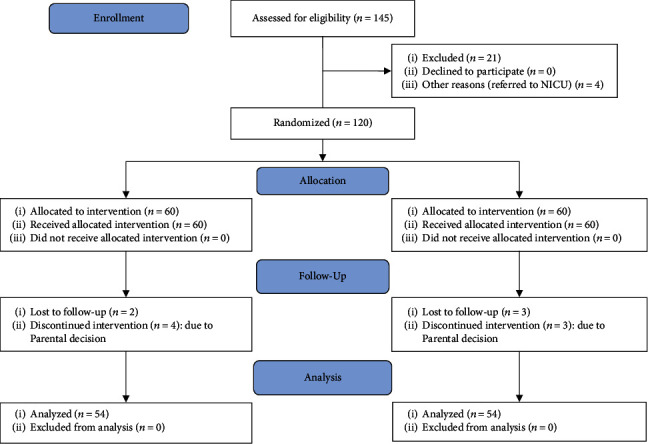
CONSORT flow diagram.

In each group, six patients lost to follow-up or discontinued intervention. At the end, fifty-four neonates in each group completed the study and their data were analyzed (Figure 1).

The primary outcomes were changes in the total number of GERD-related signs and symptoms from baseline to the end of the intervention. The secondary outcomes were defined as complications in either group following oral administration of metoclopramide, ranitidine, and lansoprazole.

### 2.4. Data Analysis

The SPSS for Windows version 21.0 was used for data analysis (SPSS Inc., Chicago, IL, USA). Descriptive data are reported as mean and standard deviation (SD) for numerical and number (percent) for categorical data. Posttreatment results were compared against baseline data using a two-sided paired *t*-test for differences in the mean values and chi-square test and Fisher's exact test (two sided) for differences in the percentage of response to treatment. A *p* value of <0.05 was considered significant.

## 3. Results

In this double-blind randomized controlled trial, 108 term newborns (mean age: 10.91 ± 7.17 days, range: 1-29 days, girls: 57.4%) were enrolled. The mean birth weight of the study subjects was 3204.5 ± 399.6 g. All participants were evaluated by the attending neonatologist and diagnosed with GERD based on the clinical criteria of the I-GERQ-R and validity score. The neonates were randomized to receive metoclopramide + ranitidine (*n* = 54, ranitidine was administered at a dose of 2 mg/kg/dose three times daily) or metoclopramide + lansoprazole (*n* = 54, lansoprazole was administered at a dose of 0.5 mg/kg/dose twice daily). There was no significant difference in demographic data and baseline characteristics between the two groups (Table 2).

Pre- and postintervention GERD-associated clinical manifestations according to the intervention group are compared in Tables 3 and 4. In the present study, the total clinical improvement was >50% after one week and >70% after one month of intervention, as defined as a reduction in the I-GERQ-R score. The findings showed that the response rate was significantly higher in the lansoprazole plus metoclopramide group compared to the ranitidine plus metoclopramide group after one week (64.38% ± 15.07% vs. 52.14% ± 21.24%, “*p* ≤ 0.001”) and after one month (74.3% ± 23.3% vs. 88.47% ± 13.18%, “*p* ≤ 0.001”). Treatment with lansoprazole plus metoclopramide improved all clinical manifestations better than ranitidine plus metoclopramide after one week while treatment with lansoprazole plus metoclopramide improved all clinical manifestations better than ranitidine plus metoclopramide except feeding refusal, vomiting associated with irritability and lethargy, extraordinary crying, apnea or respiratory problem, and redness or cyanosis during or after feeding after one month. The response rate of the above clinical manifestations was similar in both groups.

The mean ± SD score of preintervention clinical manifestations in group A was 19.52 ± 5.18 that decreased to 9.31 ± 4.57 after one week of intervention (*p* ≤ 0.001) and 4.5 ± 4.12 after one month of intervention (*p* ≤ 0.001). The mean ± SD score of preintervention clinical manifestations in group B was 20.5 ± 4.92 that decreased to 7.44 ± 3.86 after one week of intervention (*p* ≤ 0.001) and 2.41 ± 3.06 after one month of intervention (*p* ≤ 0.001) (Table 5).

## 4. Discussion

The present randomized clinical trial study was conducted to compare the efficacy and safety of oral ranitidine plus metoclopramide with oral lansoprazole plus metoclopramide in the treatment of neonatal GERD resistant to conservative therapy and monotherapy.

Oral PPIs have been increasingly used in children and infants under one year of age for treatment of GERD despite lack of published evidence for improved outcomes and increasing concerns over adverse effects [21, 22].

PPIs have earned a global approval leading to less therapy disruption and less therapy changes in the first month of treatment [8, 22]. The previous approach to the management of infantile GERD requiring acid suppression treatment was a “step-up” regimen in which ranitidine was administered as the first line of treatment and replaced with PPIs if the symptoms persisted despite using high-dose ranitidine [23]. According to an updated review on GERD in children, pharmacotherapy should be considered in the treatment of severe gastroesophageal reflux disease for patients who do not respond to conservative therapies. On the other hand, PPIs are favored over H2-receptor antagonists because of their superior efficacy [24]. Recent studies have demonstrated that the majority of symptoms in neonatal GERD are associated either with nonacid reflux or with acid reflux [25]. Despite the information presented above, PPIs have been rarely used as the first-line therapy for GERD treatment in infants and neonates due to few comparative studies versus H2RA.

To the best of our knowledge, very few clinical trials have compared the efficacy of H2RAs with PPIs and different PPIs with each other in pediatric GERD, especially in neonates and infants [16, 17]. There are also few studies that have surveyed the safety of lansoprazole in the treatment of GERD in pediatrics and infants [18, 26, 27].

Tolia et al. evaluated the safety of lansoprazole in the treatment of GERD in patients 1–12 years of age during 8–12 weeks. They administered two doses of lansoprazole including 15 mg once daily in patients ≤ 30 kg and 30 mg once daily in patients > 30 kg (mean dose 0.9 mg/kg). No serious adverse effects were found related to lansoprazole. There were only few drug-related adverse effects after dose increment. Overall, their study showed that administration of lansoprazole was safe in children 1–12 years of age and well tolerated for 8–12 weeks [26].

Springer et al. found that lansoprazole was well tolerated after 5 days of administration and increased the intragastric pH in infants less than one year of age. A decrease in the frequency of GERD symptoms was also observed [18].

Khoshoo et al. studied the clinical efficacy of two dosing regimens of lansoprazole in infants of 3-7 months with GERD diagnosed using I-GERQ-R scores of ≥16. The patients were randomly assigned to receive lansoprazole either 15 mg QD (group A) or 7.5 mg BD (group B). The control group received an extensively hydrolyzed formula (group C). The I-GERQ-R score decreased by 33% in group A versus 67% in group B (*p* < 0.05). After two weeks of treatment, both groups showed a similar significant response rate (60% versus 67%) that was higher than group C (20%). Although there was no difference in the clinical response rate of administering two dosing regimens of lansoprazole in infants with GERD, the twice-daily regimen showed a faster clinical response [27].

Among prokinetics, metoclopramide is a safe prokinetic if it is administered in a low-dose amount and a short-duration course [12]. As other prokinetics, metoclopramide increases the LES tone and gastric emptying [11].

Since no study has compared the efficacy and safety of metoclopramide plus ranitidine with metoclopramide plus lansoprazole in the management of neonatal GERD resistant to conservative therapy and monotherapy, this study was carried out.

In the present study, the total clinical improvement was >50% after one week and >70% after one month of intervention as defined as “a reduction in the I-GERQ-R score.” The findings showed that the response rate was significantly higher in the lansoprazole plus metoclopramide group compared to the ranitidine plus metoclopramide group after one week (64.38% ± 15.07% vs. 52.14% ± 21.24%, *p* ≤ 0.001) and after one month of intervention (74.3% ± 23.3% vs. 88.47% ± 13.18%, *p* ≤ 0.001). Treatment with lansoprazole plus metoclopramide improved all clinical manifestations better than ranitidine plus metoclopramide after one week while treatment with lansoprazole plus metoclopramide improved all clinical manifestations better than ranitidine plus metoclopramide except for feeding refusal, vomiting associated with irritability and lethargy, extraordinary crying, apnea or respiratory problems, and redness or cyanosis during or after feeding after one month. The response rate of the above clinical manifestations was similar in both groups.

In the present study, the neonates with GERD showed a higher response rate to the combination of an acid suppressant and metoclopramide in comparison with monotherapy alone. It seems that the combination of an acid suppressant with metoclopramide had a cumulative hypertonic effect on the lower esophageal sphincter and a more rapid gastric emptying effect that led to a higher response rate in these patients.

There are some reports that acid suppressants may induce higher infection rates, necrotizing enterocolitis and mortality in premature infants [28–30]. According to a study of Kierkus et al. [31], PPIs are well tolerated in short-term use and are associated with mild to moderate side effects. However, more studies should be done to determine the efficacy and safety of acid suppressants in infants [22]. Metoclopramide may induce irritability, drowsiness, dystonic reaction, apnea, and emesis in infants [32]. In the present study, no adverse effects were observed for drugs used in each group.

This study had some limitations. It was limited to a healthy term population, so we suggest similar studies on term infants with more participants and premature infants too. The study was limited to 30 days and no long-term follow-up of the infants was reported. More researches on longer duration of intervention and longer-term outcomes are needed before firm conclusions can be reached. Future studies with a control group are also recommended.

## 5. Conclusions

The response rate was significant in each group after one week and one month of treatment, but it was significantly higher in the “lansoprazole plus metoclopramide” group compared with the “ranitidine plus metoclopramide” group. The combination of each acid suppressant with metoclopramide led to a higher response rate in comparison with monotherapy used before intervention.

## Figures and Tables

**Table 1:** Correlations between daily symptom diary and the I-GERQ-R.

I-GERQ-R items	Scoring
Daily symptom diary	
Item 1. How often did baby spit up?	0-3
Item 2. How much did baby spit up?	0-4
Item 4. How often was spitting up uncomfortable for the baby?	0-4
Item 5. How often did the baby refuse a feeding when hungry?	0-4
Item 6. How often did the baby stop eating soon after eating even when hungry?	0-4
Item 8. Did the baby cry a lot during or within 1 hour after feedings?	0-4
Item 9. Did the baby cry or fuss more than usual?	0-4
Item 10. On average how long did the baby cry or fuss during a 24-hour period?	0-3
Item 11. How often did the baby have hiccups?	0-4
Item 12. How often did the baby have episodes of arching back?	0-4
Item 13. Has the baby stopped breathing while awake or struggled to breathe?	0-4
Item 14. Has the baby turned blue or purple?	0-2

Regurgitation and crying items comprise ~50% of total possible points; >> needed for diagnosis. Total possible scoring: 42 (22); cut point > 15.

**Table 2:** Demographic characteristics in two intervention groups.

Demographic characteristics	Ranitidine plus metoclopramide (group A) *n* = 54	Lansoprazole plus metoclopramide (group B) *n* = 54	*p* value
Gender			
Girls, *n* (%)	31 (57.4%)	28 (51.9%)	0.564
Boys, *n* (%)	23 (42.6%)	26 (48.1%)	
Age at intervention, mean ± SD, days	11.6 ± 7.48	10.22 ± 6.861	0.321
Birth weight, mean ± SD, g	3189.5 ± 428.1	3219.5 ± 371.1	0.474
Weight at presentation, mean ± SD, g	3193.6 ± 516.4	3205.8 ± 532.4	0.835
Gestational age at birth, mean ± SD, weeks ± days	38 w ± 3 d	38 w ± 5 d	0.216
Total scoring at presentation, mean ± SD	19.48 ± 5.35	20.35 ± 4.88	0.263

**Table 3:** GERD-related clinical manifestation scoring, before and one week after intervention.

GERD clinical manifestations	Mean scoring ± SD preintervention		*p* value	Mean scoring ± SD one week after intervention		*p* value
	Ranitidine plus metoclopramide (group A) *n* = 54	Lansoprazole plus metoclopramide (group B) *n* = 54		Ranitidine plus metoclopramide (group A) *n* = 54	Lansoprazole plus metoclopramide (group B) *n* = 54	
Weight	3193.6 ± 516.4	3205.8 ± 532.4	0.835	3617 ± 578.9	3606.7 ± 638.1	0.712
Vomiting intensity	1.85 ± 1.05	1.81 ± 1.01	0.835	1.19 ± 0.83	0.87 ± 0.78	0.041
Vomiting volume	2.26 ± 0.49	2.24 ± 1.24	0.912	1.20 ± 1.03	0.98 ± 1.11	0.288
Refusal feeding	1.46 ± 0.91	1.52 ± 1.3	0.781	0.83 ± 0.69	0.69 ± 0.8	0.332
Refusal feeding contrary being hungry	1.52 ± 0.93	1.54 ± 1.3	0.714	0.83 ± 0.69	0.67 ± 0.75	0.251
Crying before and after feeding	1.89 ± 1.27	1.59 ± 1.22	0.219	1 ± 0.87	0.69 ± 0.72	0.046
Crying duration	1.46 ± 0.82	1.3 ± 0.96	0.360	0.87 ± 0.67	0.67 ± 0.61	0.162
Hiccup	2.24 ± 1.13	1.91 ± 1.31	0.142	1.15 ± 0.74	0.96 ± 0.89	0.076
Arching	3.63 ± 0.7	3.87 ± 0.47	0.084	1.89 ± 0.95	1.63 ± 0.85	0.137
Vomiting associated with irritability and lethargy	1.85 ± 2.01	2.3 ± 1.99	0.248	0.74 ± 0.54	0	≤0.001
Extraordinary crying	1.19 ± 1.84	0.815 ± 1.63	0.268	0.148 ± 0.76	0	0.153
Apnea or respiratory problem	0.296 ± 0.72	0.7 ± 0.96	0.015	0.148 ± 0.53	0.074 ± 0.38	0.401
Redness or cyanosis during or after feeding	0.259 ± 0.68	0.59 ± 0.92	0.034	0.037 ± 0.27	0.037 ± 0.27	1
Total scoring	19.52 ± 5.18	20.5 ± 4.92	0.263	9.31 ± 4.57	7.44 ± 3.86	0.018

**Table 4:** GERD-related clinical manifestation scoring, before and one month after intervention.

GERD clinical manifestations	Mean scoring ± SD preintervention		*p* value	Mean scoring ± SD one month after intervention		*p* value
	Ranitidine plus metoclopramide (group A) *n* = 54	Lansoprazole plus metoclopramide (group B) *n* = 54		Ranitidine plus metoclopramide (group A) *n* = 54	Lansoprazole plus metoclopramide (group B) *n* = 54	
Weight	3193.6 ± 516.4	3205.8 ± 532.4	0.835	4465.9 ± 766.2	4372.1 ± 705.3	0.324
Vomiting intensity	1.85 ± 1.05	1.81 ± 1.01	0.835	0.60 ± 0.83	0.25 ± 0.43	0.006
Vomiting volume	2.26 ± 0.490	2.24 ± 1.24	0.912	0.62 ± 0.9	0.7 ± 1.2	0.695
Refusal feeding	1.46 ± 0.91	1.52 ± 1.3	0.781	0.42 ± 0.61	0.21 ± 0.45	0.044
Refusal feeding contrary being hungry	1.52 ± 0.93	1.54 ± 1.3	0.714	0.40 ± 0.61	0.19 ± 0.44	0.032
Crying before and after feeding	1.89 ± 1.27	1.59 ± 1.22	0.219	0.33 ± 0.59	0.15 ± 0.5	0.09
Crying duration	1.46 ± 0.82	1.3 ± 0.96	0.360	0.32 ± 0.51	0.15 ± 0.41	0.058
Hiccup	2.24 ± 1.13	1.91 ± 1.31	0.142	0.70 ± 0.54	0.57 ± 0.87	0.353
Arching	3.63 ± 0.7	3.87 ± 0.47	0.084	0.78 ± 0.46	0.53 ± 0.61	0.018
Vomiting associated with irritability and lethargy	1.85 ± 2.01	2.3 ± 1.99	0.248	0	0	—
Extraordinary crying	1.19 ± 1.84	0.815 ± 1.63	0.268	0.074 ± 0.544	0.074 ± 0.544	1
Apnea or respiratory problem	0.296 ± 0.72	0.7 ± 0.96	0.015	0.037 ± 0.27	0.037 ± 0.27	1
Redness or cyanosis during or after feeding	0.259 ± 0.68	0.59 ± 0.92	0.034	0	0.037 ± 0.27	0.315
Total scoring	19.52 ± 5.18	20.5 ± 4.92	0.263	4.5 ± 4.12	2.41 ± 3.06	0.003

**Table 5:** The changes of GERD-related scoring of clinical manifestations and percentage of response rate one week and one month after intervention.

The mean response rate	Ranitidine plus metoclopramide (group A) *n* = 54	Lansoprazole plus metoclopramide (group B) *n* = 54	Intergroup ^∗∗^*p* value
Preintervention clinical manifestation scoring, mean ± SD	19.52 ± 5.18	20.5 ± 4.92	0.263
Overall clinical manifestation scoring, one week after intervention, mean ± SD	9.31 ± 4.57	7.44 ± 3.86	0.018
Percentage of response rate one week after intervention, mean ± SD	52.14 ± 21.24	64.38 ± 15.07	
Overall clinical manifestation scoring, one month after intervention, mean ± SD	4.5 ± 4.12	2.41 ± 3.06	0.003
Percentage of response rate, one month after intervention, mean ± SD	74.3 ± 23.3	88.47 ± 13.18	
Intragroup ^∗^*p* value	≤0.001	≤0.001	≤0.001

^∗^Intragroup *p* value means *p* value between pre- and postintervention in each group; ^∗∗^intergroup *p* value means *p* value between two groups of intervention.

## Data Availability

The data used to support the findings of this study are available from the corresponding author upon request.
